# Rapid identification of bacteria and *candida* using pna-fish from blood and peritoneal fluid cultures: a retrospective clinical study

**DOI:** 10.1186/1476-0711-12-2

**Published:** 2013-01-07

**Authors:** Dana M Harris, D Jane Hata

**Affiliations:** 1 Division of Hospital Internal Medicine, Mayo Clinic College of Medicine, 4500 San Pablo Rd, Jacksonville, Florida, 32224, USA; 2 Department of Laboratory Medicine and Pathology, Mayo Clinic College of Medicine, 4500 San Pablo Rd, Jacksonville, Florida, 32224, USA

**Keywords:** PNA-FISH, Blood culture, Bacteremia, Fungemia, Peritoneal fluid

## Abstract

**Background:**

Peptide nucleic acid fluorescent *in situ* hybridization (PNA-FISH) is a rapid and established method for identification of *Candida* sp., Gram positive, and Gram negative bacteria from positive blood cultures. This study reports clinical experience in the evaluation of 103 positive blood cultures and 17 positive peritoneal fluid cultures from 120 patients using PNA-FISH. Our study provides evidence as to potential pharmaceutical cost savings based on rapid pathogen identification, in addition to the novel application of PNA-FISH to peritoneal fluid specimens.

**Methods:**

Identification accuracy and elapsed time to identification of Gram positives, Gram negatives, and *Candida* sp., isolated from blood and peritoneal fluid cultures were assessed using PNA-FISH (AdvanDx), as compared to standard culture methods. Patient charts were reviewed to extrapolate potential pharmaceutical cost savings due to adjustment of antimicrobial or antifungal therapy, based on identification by PNA-FISH.

**Results:**

In blood cultures, time to identification by standard culture methods for bacteria and *Candida* sp., averaged 83.6 hours (95% CI 56.7 to 110.5). Identification by PNA-FISH averaged 11.2 hours (95% CI 4.8 to 17.6). Overall PNA-FISH identification accuracy was 98.8% (83/84, 95% CI 93.5% to 99.9%) as compared to culture. In peritoneal fluid, identification of bacteria by culture averaged 87.4 hours (95% CI −92.4 to 267.1). Identification by PNA-FISH averaged 16.4 hours (95% CI −57.3 to 90.0). Overall PNA-FISH identification accuracy was 100% (13/13, 95% CI 75.3% to 100%). For *Candida* sp., pharmaceutical cost savings based on PNA-FISH identification could be $377.74/day. For coagulase-negative staphylococcus (CoNS), discontinuation of vancomycin could result in savings of $20.00/day.

**Conclusions:**

In this retrospective study, excellent accuracy of PNA-FISH in blood and peritoneal fluids with reduced time to identification was observed, as compared to conventional culture-based techniques. Species-level identification based on PNA-FISH could contribute to notable cost savings due to adjustments in empiric antimicrobial or antifungal therapy as appropriate to the pathogen identified.

## Background

Rapid identification of pathogens in blood and peritoneal fluid plays a critical role in patient outcomes, and healthcare cost. Bloodstream associated infections (BSI) are the 10^th^ leading cause of death in the U.S [1]; this rate has increased by 78% over the last 2 decades**.** Bloodstream infections acquired in hospitals account for approximately 250,000 cases each year with a direct attributable mortality rate of 16-40%. Total hospitalization time is prolonged by 4.5-32 days [2], and inappropriate empirical antimicrobial therapy is an important predictor of death in this patient population [3].

In identification of organisms implicated in BSI, automated blood culture followed by biochemical analysis of isolates is considered the gold standard [4]. New detection technologies for organism identification from blood culture such as real-time PCR, DNA microarrays, matrix-assisted laser desorption ionization time of flight mass spectrometry (MALDI-TOF), and peptide nucleic acid fluorescent *in situ* hybridization (PNA-FISH) for detection of *Candida*, Gram positive, and Gram negative organisms may increase organism identification accuracy, and significantly reduce time to result [5].

PNA-FISH is a U.S. Food and Drug Administration (FDA) approved commercially available method for the detection of bacteria and yeast species directly from positive blood culture bottles. This methodology utilizes hybridization of PNA probes to organism-specific rRNA, with detection via fluorescent microscopy [6,7]. To date, studies utilizing PNA-FISH have been reported for detection of Gram positive organisms, *Candida*, and other Gram negative species [3,8-12]. Although not specifically FDA-approved for this indication, our study also assessed identification accuracy from peritoneal fluid inoculated into blood culture media. We are unaware of other published studies evaluating PNA-FISH for identifying organisms from peritoneal fluid.

The purpose of our study was to compare PNA-FISH to traditional culture techniques in blood and peritoneal fluids for accuracy in identification to species, and overall time to identification of Gram positive organisms, Gram negative organisms and *Candida* spp. In a secondary analysis, we specifically examined the potential pharmaceutical cost savings when the correct species of *Candida* was identified with concomitant correction of antifungal therapy as necessary. This manner of analysis was also applied to vancomycin costs when identification of coagulase-negative *Staphylococcus* (CoNS) was achieved by PNA-FISH in blood cultures.

## Methods

This study was approved by the Mayo Clinic Institutional Review Board. This study retrospectively evaluated blood or peritoneal fluid cultures from 120 patients routinely submitted to the Clinical Microbiology Laboratory at Mayo Clinic in Jacksonville, FL from February through June 2009. Testing was performed using both traditional blood culture methodology and PNA-FISH (AdvanDx, Woburn, MA). PNA-FISH testing and standard blood culture workup was performed by two different technologists, with reciprocal blinding of results.

### Blood/peritoneal fluid cultures

Blood cultures and peritoneal fluid cultures were performed using BACTEC Plus Aerobic F and Plus Anaerobic F bottles (Becton Dickinson, Sparks, MD) using standard methods and according to the manufacturer’s recommendations [4]. As part of a routine culture set consisting of one aerobic and one anaerobic bottle, 8.0 to 10.0 mL of blood or peritoneal fluid were inoculated in each BACTEC bottle and incubated at 37°C. When the first bottle of the pair signaled positive by the BACTEC 9240 instrument and confirmed by Gram stain, an aliquot from the bottle was subcultured to a blood agar plate and incubated at 37°C. After sufficient pure bacterial growth was achieved (24 – 88 hours), identification tests were performed on a Microscan Walkaway-96 system utilizing a 24 hour incubation period using GP-33 or GN-34 combination panels as appropriate (Siemens Healthcare Diagnostics, Deerfield IL).

### PNA-FISH

Blood culture bottles signaling positive in the BACTEC system were tested on the same day with PNA-FISH. Based on Gram stain results from the signal-positive blood culture bottle, a specific PNA-FISH probe was selected for use. These consisted of the *S. aureus*/CoNS, E*. faecalis*/OE (other Enterococci, *E. faecium*), *E. coli/P. aeruginosa*, EK (*E. coli, K. pneumoniae*/*P. aeruginosa)*. For yeasts, probes for *C. albicans/C. glabrata,* and the Yeast Traffic Light (*C. albicans/C. parapsilosis* - green, *C. glabrata/C. krusei* - red, *C. tropicalis* – yellow) were utilized. The PNA-FISH method was performed according to manufacturer’s recommendations. One drop of fixation solution was gently mixed with one drop (10 μl) of specimen from a positive BACTEC bottle on a PNA-FISH slide. Slides were fixed in methanol, followed by immersion in 80% ethanol for 10 minutes. Slides were allowed to air dry. One drop of specific PNA probe mixture was applied to the slide, a cover slip was applied, and the slide was hybridized for 90 minutes at 55°C. Post-hybridization, slides were incubated in 55°C wash solution for 30 minutes. Slides were allowed to air dry, and mounted. Slides were examined at 60X on an Olympus BX-41 fluorescent microscope equipped with a 528-633λ dual band filter. Slides were examined for the presence of multiple bright fluorescent (green, red, yellow), morphologically consistent microorganisms in multiple fields of view. Organisms resembling yeast were initially tested with the *C. albicans*/*C. glabrata* PNA-FISH probe. If negative, PNA-FISH was repeated with the Yeast Traffic Light probe in order to identify presence of *C. parapsilosis*, *C. krusei*, or *C. tropicalis*. Gram-negative organisms were initially tested with the *E. coli/P. aeruginosa* probe. If negative, PNA-FISH was repeated with the EK/*P. aeruginosa* probe in order to identify presence of *Klebsiella pneumoniae.*

Probe-specific PNA-FISH positive and negative control slides were obtained from the manufacturer and utilized for quality control each time PNA-FISH was performed. All bacterial and yeast identifications from blood and peritoneal fluid cultures were confirmed using standard methods by the Clinical Microbiology Laboratory at Mayo Clinic in Jacksonville, FL.

Identification accuracy and time to identification using both PNA-FISH and culture (gold standard) were assessed. Time to identification with culture (C-ID) was calculated from the time the BACTEC bottle signaled positive, to the time at which the microbiology laboratory identified the organism. Time to identification with PNA-FISH (F-ID) was calculated from the time the BACTEC bottle initially signaled positive, to completion of F-ID. Appropriate controls were performed with each PNA-FISH test batch. PNA-FISH was performed in batches twice daily at 8am and 1pm. All samples that became BACTEC positive between those times were held until the next processing period, and the time elapsed until processing was added to the total F-ID time. Time saved was defined as the difference between C-ID and F-ID. Organisms that could not be identified by PNA-FISH were not included in C-ID or F-ID calculations.

In order to better assess detection and identification of *Candida* spp., 15 BACTEC bottles were spiked (9 blood, 6 peritoneal fluid) by mixing 8.0 mL of culture-negative blood or peritoneal fluid with 2.0 mL of a 0.5 McFarland suspension of a known species of yeast and inoculating the BACTEC bottle. Bottles were incubated under standard conditions until they signaled positive. At that time the bottle was removed from the BACTEC instrument and processed as previously described. The results from these spiked bottles were included only in the accuracy calculations, and not calculated time to detection. Statistical significance of time to C-ID versus time to F-ID was calculated using t test method and GraphPad [13].

Patient charts were reviewed for selection of empiric antimicrobial in order to extrapolate potential pharmaceutical cost savings of PNA-FISH. Pharmaceutical costs were based on the 2009 institutional Average Wholesale Price (AWP). The AWP for oral fluconazole 400 mg daily was $27.26, and caspofungin 50 mg daily was $405.00. The AWP of IV vancomycin 2 grams daily was $20.00 [14].

## Results

**Figure 1:**
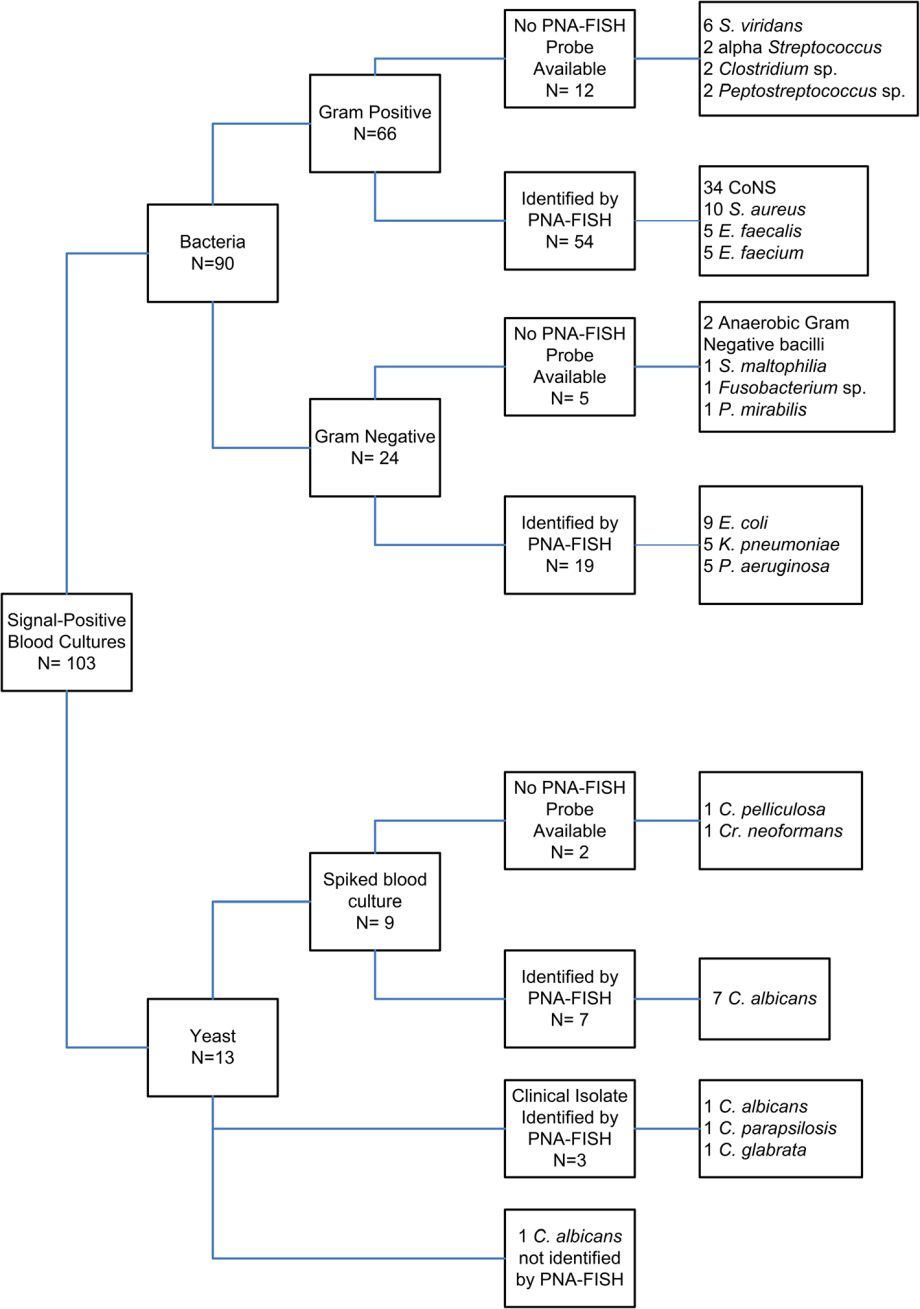
Analysis of Blood Culture by PNA-FISH.

**Figure 2:**
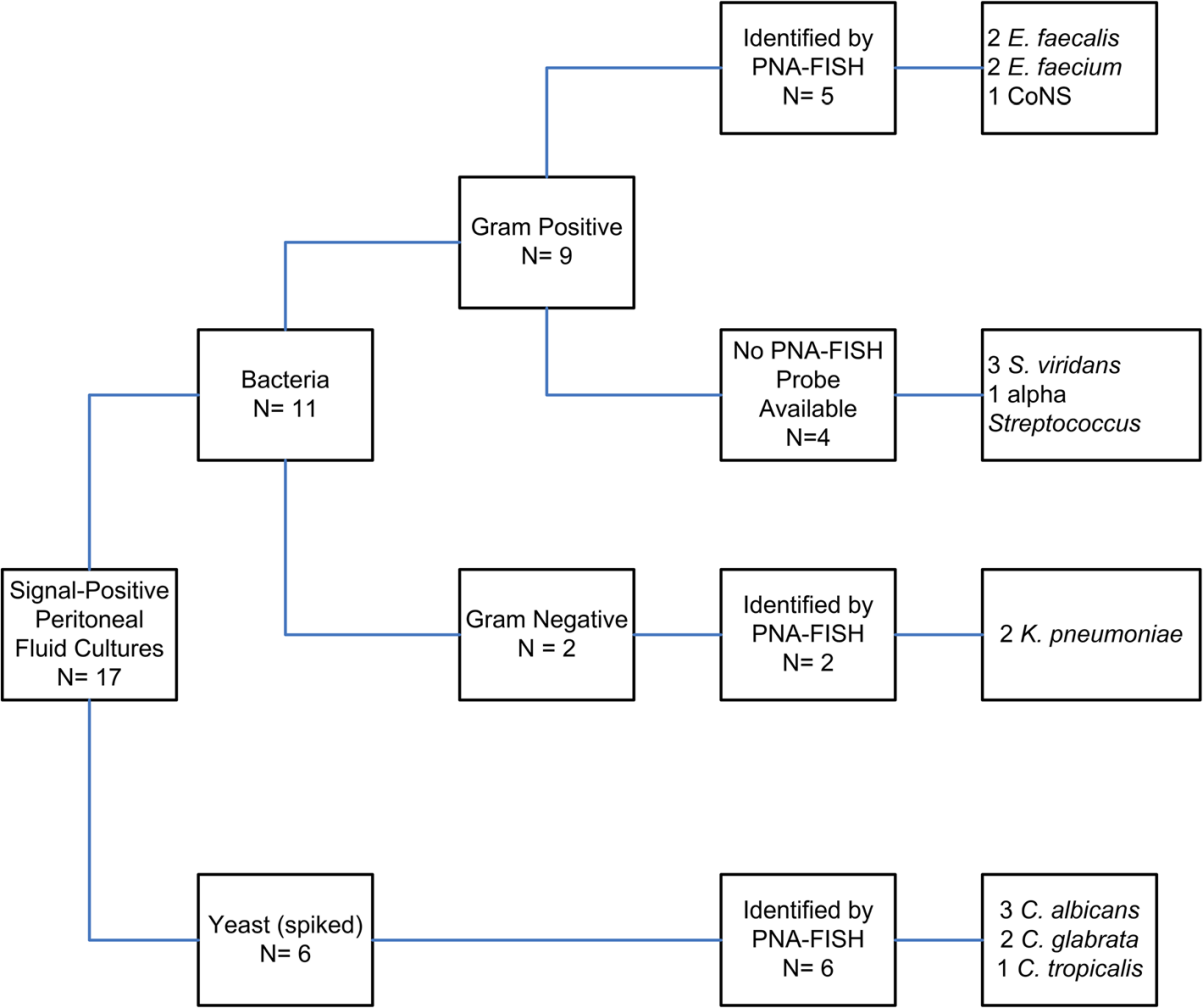
Analysis of Peritoneal Fluid Cultures by PNA-FISH.

A total of 103 blood culture bottles and 17 peritoneal fluid culture bottles were signal-positive for bacterial or fungal growth after incubation in the BACTEC system. Of those, 9 blood and 6 peritoneal fluid culture bottles had been spiked with *Candida* sp. or other yeasts in the laboratory in order to increase sample numbers to statistically meaningful levels specific for the pathogen. Compared to traditional culture methods, 96 of 120 positive BACTEC blood culture bottles contained an organism for which a PNA-FISH probe was available. Twenty-three signal-positive bottles contained a bacteria or yeast for which a PNA-FISH probe was not available [Figure 1, Figure 2]. All 23 of these bottles tested negative by PNA-FISH probes used in this study.

In signal-positive blood culture bottles containing Gram positive bacteria, the following species were identified by PNA-FISH: 34 coagulase-negative *Staphylococcus* [CoNS], 10 *S. aureus*, 5 *E. faecalis*, 5 *E. faecium*. In signal-positive blood culture bottles containing Gram negative bacteria, the following species were identified: 9 *E. coli*, 5 *K. pneumoniae*, 5 *P. aeruginosa.* In signal positive blood culture bottles containing yeasts, the following species were identified: 8 *C. albicans,* 1 *C. glabrata,* 1 *C. parapsilosis.* One isolate from a positive blood culture bottle confirmed as *C. albicans* reacted only weakly with the *C. albicans*/*C. glabrata* dual probe as well as the PNA-FISH Traffic Light, and was judged to be negative by PNA-FISH. Of the 9 blood culture bottles spiked with yeasts; 7 positive bottles containing *C. albicans* were correctly identified by PNA-FISH. Two spiked bottles contained a yeast for which a PNA-FISH probe was not available (*C. pelliculosa*, *Cr. neoformans*); both bottles were negative with the yeast dual probe and Traffic Light probe [Figure 1]. Spiked bottles were included for accuracy calculations, but not included in calculation of time to results.

Compared to conventional blood culture identification, Gram positive organisms were identified 70.2 hours faster with PNA-FISH (95% CI 60.2 to 80.3, p< 0.0001) and Gram negative organisms were identified 64.1 hours more rapidly (95% CI 51.0 to 77.2, p =0.0002). *Candida* sp. were identified 83.0 hours faster with PNA-FISH compared to culture (95% CI 33.8 to 132.3 p=0.0095) [Table 1]. For bacteria, overall accuracy of identification with PNA-FISH compared to culture was 100% (73/73, 95% CI 95% to 100%). Overall accuracy of *Candida* sp. identification was 90.9% (10/11, 95% CI 58.7% to 99%).

**Table 1:** Time to Identification of isolates from blood and peritoneal fluids by culture and PNA-FISH

**BLOOD CULTURES**					
**Organism**	**N**	**Mean Culture Hours to ID (range)**	**Mean PNA-FISH Hours to ID (range)**	**Mean Hours Saved with PNA-FISH (range)**	***P*(Time Saved)**
All Gram Positive	54	78.5 h	8.3 h	70.2 h	<0.0001
		(20–112)	(3–22)	(1 – 94)	
CoNS	34	72.2	12.5 h	59.7 h	<0.0001
		(20–103)	(3 – 22)	(1 – 93)	
*S. aureus*	10	79.0 h	9.9 h	65.8 h	<0.0001
		(47 – 98)	(3–20)	(33 – 93)	
*E. faecalis*	5	73.8 h	5.8 h	68.0 h	<0.0001
		(60 – 97)	(3 – 15)	(45 – 94)	
Other Enterococci (includes *E. faecium*)	5	89.0 h	6.4 h	82.6 h	<0.0001
		(70 –112)	(3 – 20)	(69 – 93)	
All Gram Negative	19	76.2 h	12.1 h	64.1 h	0.0002
		(23 –112)	(3 – 24)	(3–115)	
*E. coli*	9	78.4 h	9.2 h	69.4 h	<0.0001
		(25 –108)	(5 – 18)	(20 – 91)	
*K. pneumoniae*	5	71.6 h	17.8 h	54.0 h	0.0109
		(23 –112)	(5 – 24)	(3 – 91)	
*P. aeruginosa*	5	70.0 h	7.8 h	58.8 h	0.0002
		(40–94)	(3 – 22)	(18 – 90)	
All *Candida*	4	96.0 h	13.2 h	83.0 h	0.0095
		(72 –130)	(7.2 – 17.2)	(65 – 115)	
*C. albicans*	2*	72.0 h	7.2 h	64.8 h	N/A
*C. glabrata*	1	130 h	15.2 h	114.8 h	N/A
*C. parapsilosis*	1	86	17.2 h	68.8 h	NA
**PERITONEAL FLUID CULTURES**^**†**^					
All Gram Positive	5	73.2 h	10.6 h	62.6 h	0.0070
		(23–111)	(2 – 22.2)	(21 – 90.8)	
CoNS	1	111.0 h	19.6 h	91.4 h	N/A
*E. faecalis*	2	35.0 h	4.5 h	30.5	0.1306^‡^
		(23–47)	(2 – 7)	(21 – 40)	
Other Enterococci (includes *E. faecium*)	2	92.5 h	12.1 h	80.h	0.0522^‡^
		(75 – 110)	(5 – 20)	(70 – 90)	
All Gram Negative	2	101.5 h	22.2 h	79.3 h	0.0204
*K. pneumoniae*	2	101.5 h	22.2 h	79.3 h	0.0204
		(90 – 113)	(22.2)	(67.8 – 90.8)	

* One isolate not identified by PNA-FISH.

^**†**^ PNA-FISH is not FDA approved for this sample type.

^‡^ Not statistically significant.

Results from peritoneal fluid cultures were analyzed separately from blood cultures due to potential differences in PNA-FISH performance due to sample type. In peritoneal fluid cultures (n=17), 11 specimens signaled positive for bacterial growth. Among signal-positive blood culture bottles containing Gram positive bacteria, the following species were identified: 2 *E. faecalis,* 2 *E. faecium,* 1 CoNS. Four bottles contained *Streptococcus* viridans, and subsequently could not be identified by PNA-FISH. Two signal-positive bottles contained Gram negative bacteria; 2/2 isolates were identified as *K. pneumoniae* by PNA-FISH. During the period of the study, no peritoneal infections with *Candida* sp. were noted among clinical specimens submitted to the laboratory. Among the 6 peritoneal fluid bottles spiked with *Candida* sp. (3 *C. albicans*, 2 *C. glabrata*, 1 *C. tropicalis*) all species were correctly identified by PNA-FISH [Figure 2]. Results from spiked bottles were included only in the accuracy calculation; comparative time to detection was not calculated. Gram positive organisms were identified 62.6 hours faster with PNA-FISH compared to culture (95% CI 2.8 to 132.1, p=0.0070) and Gram negative organisms were identified 79.3 hours faster with PNA-FISH (95% CI 29.82 to 128.78, p=0.0204) [Table 1]. Overall mean time saved using PNA-FISH for detection of Gram positive and Gram negative organisms in peritoneal fluid cultures was 70.95 hours (95% CI 5.2 to 136.7, p=0.0435). The overall accuracy for detection of bacteria and *Candida* sp. in peritoneal fluid was 100% (6/6, 95% CI 54% to 100%). During testing of blood and peritoneal fluids, all PNA-FISH control slides performed as expected, thus the inter-run precision of the test was 100%.

### Projected pharmaceutical costs

Antifungal/antimicrobial costs were extrapolated based on organism identification from blood, time to identification, and chart review of therapy [Table 2]. In 2 of 3 patients in which C-ID, F-ID, and chart review was available, two patients were empirically treated with caspofungin for suspected *C. glabrata* fungemia. When identification was finalized by conventional methods as *C. albicans* and *C. parapsilosis*, therapy was changed to fluconazole. Using PNA-FISH, identification of *C. albicans* and *C. parapsilosis* could have been achieved in an average of 1/2 day, compared to 4 days with C-ID, resulting in potential antifungal cost savings due to change of therapy from caspofungin to fluconazole of $377.74 per day.

**Table 2:** Potential pharmaceutical cost savings with PNA-FISH

**Case**	**Final Species ID**	**Initial Rx**	**Rx initiation until change (days)**	**Time to culture ID (days)**	**Time to PNA-FISH ID (days)**	**Potential time saved w/PNA-FISH (days)**	**Potential cost saved w/PNA-FISH**^**†**^
1	*C. albicans*	Caspofungin	6	3	0.3	2.7	$1,093.50
2	*C. glabrata*	Fluconazole	2	5.4	0.6	4.8	Fluconazole changed to Caspofungin
3	*C. parapsilosis*	Caspofungin	5	3.6	0.7	2.9	$1,174.50
1	CoNS	Vancomycin	3	3.7	0.8	2.9	$58.00
2	CoNS	Vancomycin	4	2.1	0.2	1.9	$38.00
3	CoNS	Vancomycin	5	3.2	0.3	2.9	$58.00
4	CoNS	Vancomycin	2	0.8	0.7	0.1	$2.00

^†^ Potential time saved w/PNA-FISH X AWP = Potential cost saved w/PNA-FISH.

AWP:

Fluconazole 400mg po/day= $27.26.

Caspofungin 50mg IV/day=$405.00.

Vancomycin 2 g/day=$20.00.

Four patients were selected for chart review in order to determine the potential effect of time to culture result on antibiotic costs. Each case tested positive for CoNS in blood cultures by C-ID and F-ID. Upon initial positive blood culture, patients were treated with vancomycin until culture identification of CoNS by C-ID was verified. At that point, based on clinical presentation, CoNS was considered a contaminant, resulting in discontinuation of vancomycin therapy. The time to result by C-ID was compared to the time to result by F-ID, and potential time to result saved by F-ID calculated. In this small subset of patients, the use of PNA-FISH, for rapid identification of CoNS, could have resulted in discontinuation of IV vancomycin an average of 2.2 days earlier, with an estimated savings of $20.00/day [Table 2].

## Discussion

PNA-FISH has been shown to be a useful and accurate method in the identification of Gram positive, Gram negative, and *Candida* sp. from blood cultures and peritoneal fluid cultures. When analyzing PNA-FISH probe performance for both bacteria and yeast combined in both sample types, we demonstrated an overall identification accuracy of 98.9% (96/97). Accuracy of PNA-FISH for bacteria was 100% (80/80), accuracy for *Candida* sp. was 94.1% (16/17). This high level of accuracy with PNA-FISH has been replicated in other studies [6-10,12,15]. We did not test identification accuracy in BACTEC-negative bottles, as PNA-FISH requires an organism concentration of at least 10^5^ CFU/mL for detection.

Clinical isolates identified in this study are representative of the most commonly reported pathogens implicated in nosocomial bloodstream infections in the United States [2]. In blood cultures, accuracy of PNA-FISH for bacteria was 100%. In this study, accuracy for yeast identification was 90.9% (10/11). A single weakly reactive *C. albicans* isolate may have been due to a mismatch between organism rRNA and PNA-FISH probe secondary to point mutations in sequence, as have been described by other authors [11,12].

Mean time saved using PNA-FISH for blood cultures compared to traditional culture methods averaged 72.4 hours from the time the organism (bacteria or yeast) was detected by Gram stain from a positive blood culture bottle until final identification of species [Table 1]. The significant difference in time of C-ID as compared to F-ID may partially be attributed to conventional laboratory workflow with C-ID; after subculture of a positive blood culture bottle, plate cultures were assessed once daily for adequate growth. Panels used for automated bacterial identification were set up once daily if plate culture growth was sufficient followed by 24-hour incubation. An advantage of the PNA-FISH methodology is ease of use, in that multiple final identifications may be generated in a single laboratory shift directly from positive blood culture bottles, without need for additional incubation.

The use of blood culture media is a useful method of enhancing recovery of microorganisms from peritoneal fluids [16-18]. Although not specifically FDA-approved for this indication, we also assessed performance of PNA-FISH using peritoneal fluid specimens incubated in blood culture bottles, as proof of concept. To the best of our knowledge this study is the first application of this PNA-FISH method for this sample type. Identification accuracy for both bacteria and *Candida* sp. was 100%. Although mean time saved with PNA-FISH was not significant for *Enterococcus* sp., this was likely attributable to low sample numbers (Table 1).

Laboratory-spiked bottles were not used for turnaround time determination for *Candida* sp. in peritoneal fluids, as the intent of the study was to mimic actual clinical performance of PNA-FISH from blood culture media as much as possible. Although the turnaround time for *Candida* sp. could not be specifically determined due to the use of spiked bottles, based on blood culture data, it is reasonable to assume a similar reduction in time to F-ID in peritoneal fluid samples would also apply. Low numbers of peritoneal fluids tested is a limitation of this study. Although blood culture bottles containing peritoneal fluid spiked with *Candida* sp. were used to increase numbers to statistically meaningful levels, every attempt was made to apply PNA-FISH to situations found in actual clinical practice. Excellent performance of PNA-FISH with this sample type is encouraging for application to larger scale studies required to truly assess assay performance.

Accurate identification of pathogens is a primary driver of antimicrobial or antifungal selection. Delays in appropriate therapy clearly affect patient outcomes in a negative fashion. Among 492 intensive care patients studied by Ibrahim *et al.,* 30% received inadequate antimicrobial therapy for bacteremia; hospital mortality rate was 62% compared to 28.4% of patients receiving appropriate antibiotics [3]. In terms of yeast identification, both SENTRY and EIEIO sentinel surveillance programs demonstrated a shift in pathogenic *Candida* species over the last decade [19]. These findings support the importance of rapid identification of yeast to species level as critical to direct appropriate antifungal therapy*.* Excellent performance by PNA-FISH in both bacteria and yeast will address these clinical needs.

In this study, based on a limited chart review of clinical cases, the more expensive antifungal caspofungin was used empirically for 5–6 days in 2 cases in which the organism was *C. albicans* or *C. parapsilosis* fully susceptible to fluconazole. Based on our estimated antifungal cost savings, and 2009 AWP data, this could potentially result in pharmaceutical cost savings of $1,888.70 over 5 days of therapy. Significant pharmaceutical cost savings due to de-escalation of therapy from an echinocandin to fluconazole in infections caused by fluconazole –susceptible *C. glabrata* have also been noted in other studies [20].

We also examined the costs associated with the use of vancomycin for CoNS infection. In four cases in which chart review was available, empiric therapy with vancomycin was continued for 2–5 days and not discontinued until CoNS was identified and deemed a contaminant, based on clinical presentation of the patient. Avoiding unnecessary use of vancomycin in these instances could save an estimated $20.00 daily per patient. Based on our study, and use of PNA-FISH, more appropriate use of vancomycin and caspofungin could reduce hospital pharmaceutical costs. As indicated in other studies, rapid organism identification and judicious use of antibiotics has overall broader implications in antibiotic stewardship and may positively affect reduction of antibiotic resistance and patient mortality [6,8,21].

Based upon chart review, a disparity was noted between time of C-ID or F-ID, and the time of therapy initiation until a change in therapy was made based on ID results (Table 2). We cannot specifically comment why, in some cases, antifungal or antibiotic therapy was not changed after organism ID was finalized. This obviously affects overall estimates of pharmaceutical cost. Based on traditional practice patterns, it can only be assumed that if patients are improving under a given course of therapy, clinicians are less likely to change therapy, even with the availability of ID results. Proactive management of identification results by stakeholders such as Infectious Disease or Pharmacy, as part of an active antibiotic stewardship program will contribute to the downstream benefits of rapid diagnosis by PNA-FISH.

A limitation of PNA-FISH is a requirement of an organism concentration of at least 10^5^ CFU/mL for detection. This requirement may prove to be problematic for detection of slow-growing, or fastidious organisms. At the time this study was performed, a limited number of PNA-FISH probes were available. However, additional specific PNA-FISH probes are now available for Group B *Streptococcus* , GNR Traffic Light (FDA-approved), *C. dubliniensis, C. parapsilosis*, *K. pneumoniae*, and *Acinetobacter* (analyte-specific reagents). Recent FDA approval of a more rapid PNA-FISH protocol should prove to be advantageous in further decreasing turnaround time to organism identification [9].

## Conclusion

This study expands the body of knowledge evaluating PNA-FISH, a useful method for identification of organisms directly from blood culture bottles. Our study demonstrated excellent accuracy of identification of organisms for which PNA-FISH probes were available. This includes the most commonly isolated species implicated in bacteremia and fungemia. In addition, successful use of PNA-FISH in peritoneal fluid cultures could be extremely beneficial in analysis of this sample type. Turnaround time for final identification from both blood cultures and peritoneal fluid was significantly reduced in comparison to traditional culture methods. A recently approved shortened hybridization protocol will further contribute to a reduction in laboratory turnaround time. Although the correct choice of a PNA-FISH probe is contingent upon correct interpretation of a Gram stain, we do not feel this is an issue with properly trained laboratory technologists. PNA-FISH is easy to perform in the clinical laboratory and does not require significant capital equipment costs unlike microarrays or MALDI-TOF. PNA-FISH requires a microscope equipped with a fluorescent lamp and dual band filters for interpretation of results. Of primary importance, the accuracy and specificity of PNA-FISH can significantly affect antibiotic and antifungal utilization, allowing for more targeted therapy, reduction of duration of therapy, with an overall reduction of healthcare costs, and increased benefit to patients.

## Abbreviations

PNA-FISH: Peptide nucleic acid fluorescent *in situ* hybridization; BSI: Bloodstream associated infections; MALDI-TOF: Matrix-assisted laser desorption ionization time of flight mass spectrometry; CoNS: Coagulase-negative *Staphylococcus* ; C-ID: Time to identify with culture; F-ID: Time to identify with PNA-FISH; AWP: Average wholesale price.

## Competing interests

The authors declare that they have no competing interests.

## Authors’ contributions

DMH performed laboratory testing, data acquisition, chart review and pharmaceutical cost analysis. DJH contributed to study design, data analysis, and result interpretation. All authors contributed to the preparation of the manuscript. All authors have read and approved the final manuscript.

## Previous presentations

This study was presented in part at the 49^th^ Interscience Conference on Antimicrobial Agents and Chemotherapy, San Francisco, CA, Sept. 12 – 15, 2009
